# Nutritional Status of Flemish Vegetarians Compared with Non-Vegetarians: A Matched Samples Study

**DOI:** 10.3390/nu2070770

**Published:** 2010-07-14

**Authors:** Peter Deriemaeker, Katrien Alewaeters, Marcel Hebbelinck, Johan Lefevre, Renaat Philippaerts, Peter Clarys

**Affiliations:** 1 Department of Human Biometry and Biomechanics, Faculty of Physical Education and Physical Therapy, Vrije Universiteit Brussel, Pleinlaan 2, B-1050 Brussel, Belgium; Email: kalewaet@vub.ac.be(K.A.); mhebbel@vub.ac.be(M.H.); pclarys@vub.ac.be(P.C.); 2 Department of Biomedical Kinesiology, Faculty of Kinesiology and Rehabilitation Sciences, K.U.Leuven, Tervuursevest 101, B-3001 Leuven, Belgium; Email: Johan.Lefevre@faber.kuleuven.be; 3 Department of Movement and Sports Sciences, Ghent University, Watersportlaan 2, B-9000 Gent, Belgium; Email: Renaat.Philippaerts@UGent.be

**Keywords:** vegetarians, matched samples, nutritional status

## Abstract

The present study compares the nutritional status of vegetarian (V) with non-vegetarian (NV) subjects. A three-day food record and a health questionnaire were completed by 106 V and 106 NV matched for following characteristics: sex, age, BMI, physical activity, tobacco use and alcohol consumption. Total energy intake was not significantly different (men: V: 2,346 ± 685 kcal/d; NV: 2,628 ± 632 kcal/d; p = 0.078; women: V: 1,991 ± 539 kcal/d; NV: 1,973 ± 592 kcal/d; p = 0.849). Macronutrients intake differed significantly between the V and NV subjects for protein (men: V:12.7 ± 2.3 E%; NV:15.3 ± 4.5 E%; p = 0.003; women: V: 13.2 ± 2.3 E%; NV:16.0 ± 4.0 E%; p < 0.001), fat (men: V: 29.3 ± 8.4 E%; NV: 33.8 ± 5.3 E%; p = 0.010; women: V: 29.7 ± 6.9 E%; NV: 34.7 ± 9.0 E%; p < 0.001), and carbohydrate (men: V: 55.3 ± 10.1 E%; NV: 47.4 ± 6.9 E%; p < 0.001; women: V: 55.1 ± 7.6 E%; NV: 47.2 ± 8.2 E%; p < 0.001). The intake of most minerals was significantly different between the V and the NV subjects. V had a lower sodium intake, higher calcium, zinc, and iron intake compared to the NV subjects. Our results clearly indicate that a vegetarian diet can be adequate to sustain the nutritional demands to at least the same degree as that of omnivores. The intakes of the V subjects were closer to the recommendations for a healthy diet when compared to a group of well matched NV subjects.

## 1. Introduction

Vegetarian people do not eat meat (including fowl) or seafood, or products containing these foods. A lacto-ovo vegetarian eating pattern is based on grains, vegetables, fruits, legumes, seeds, nuts, dairy products, and eggs [1]. Epidemiological studies on vegetarians show that appropriately planned vegetarian diets are healthy and nutritionally adequate [1,2,3,4,5]. Compared to omnivorous diets, vegetarian diets can provide several health benefits [6,7,8,9]. However, these positive health-related outcomes in vegetarians can be influenced by factors other than dietary practices. A healthy life-style, including regular physical activity and the avoidance of harmful practices such as smoking and drinking, are some of the influencing factors [10]. To date, most studies compared a self-selected vegetarian group with standard population references [1]. In order to truly attribute the health advantages associated with a vegetarian life-style to the dietary pattern, it is necessary to compare the vegetarian subjects with an adequate reference sample. The matched samples principle uses samples of subjects that have been matched to eliminate individual differences and that are respectively subjected to the control and the experimental condition [11,12,13]. In such a design, every vegetarian subject is compared with a non-vegetarian subject matched for possible confounding factors.

It was the aim of this study to compare the nutritional intake between 106 vegetarians (V) and 106 matched non-vegetarians (NV). Matched samples were formed based on the following parameters: sex, age, body mass index (BMI), physical activity, tobacco use and alcohol consumption.

## 2. Methods

### 2.1. Subjects

The vegetarian subjects were recruited in the Flemish region of Belgium through advertising in health food shops, on the website of vegetarian and animal rights associations, and through word of mouth in 2005. Inclusion criteria were being a vegetarian for at least one year and being at least 20 years of age. Vegetarianism was defined as abstinence of meat, game, poultry, and fish in the diet. A total of 650 people responded, of whom 405 actually completed a health and fitness related questionnaire. Of these 405 volunteers, 375 met the inclusion criteria, corresponding to a completion rate of 58%. The male subjects ranged between 23 and 85 years of age with a mean ± standard deviation (SD) age of 42.3 ± 15.9 years and a median age of 38, while the female subjects ranged between 20 and 78 years of age with a mean ± SD age of 37.0 ± 12.3 years and a median age of 34. More women participated than men: 63.2% and 36.8%, respectively. Detailed establishment and description of this sample has been given elsewhere [14]. Of these 375 volunteers, only 106 V subjects (35 men and 71 women) completed the time consuming three-day food record. Matching with NV subjects was done based on the following characteristics: sex, age, BMI, level of physical activity, tobacco use and alcohol consumption. NV subjects were selected from the database collected by the Flemish Policy Research Centre Sport, Physical Activity and Health (SPAH) between October 2002 and April 2004. One of the aims of the SPAH study was to investigate the cross-sectional relationship between physical activity, physical fitness and several health parameters among the adult population of Flanders, the Flemish part of Belgium. Detailed establishment and description of this sample have been given elsewhere [15]. A total of 5,170 subjects volunteered to participate in the SPAH study. Only 1,520 subjects completed a three-day food record, out of which we selected proper matches for the 106 V subjects. First matching was done based on sex. Secondly, age was taken into account using seven different age categories: 18-24.99 years, 25-34.99 years, 35-44.99 years, 45-54.99 years, 55-64.99 years, 65-74.99 years, 75-84.99 years. For matching BMI, five categories were used: <18, 18.01-25, 25.01-30, 30.01-35, >35. Individual energy requirements were calculated taking into account the physical activity level (PAL), body weight and age. The next matching parameter was the smoking habits of the subjects: a yes/no classification was applied, and where necessary, the fact whether smoking subjects were light or heavy smokers was taken into account. The final matching parameter was the subject’s alcohol use, where a distinction between alcohol use during the week and alcohol use during the weekend was made. All subjects of the SPAH study and the vegetarian study signed an informed consent statement and received explanation about the purpose and procedures of the study before participating. The SPAH study was approved by the ethical and medical committee of the Katholieke Universiteit Leuven and the Ethics Committee of the Ghent University Hospital [15], and the study on vegetarians was approved by the ethical and medical committee of the Vrije Universiteit Brussel.

### 2.2. Questionnaires

For the V subjects, the self-administered questionnaire consisted of general questions on socio-economic characteristics (educational level) and health related questions concerning smoking habits and alcohol use (during week and weekend), physical activity and nutrition. Items were taken from the Belgian National Health Survey [16], which is a validated instrument that is used to estimate health related issues in the Belgian population every five years. Additional information on physical activity was collected using a Baecke questionnaire [17] to define the physical activity level (PAL) of the V subjects, since questions related to physical activity were sparse in the National Health Survey. 

For the NV subjects, the physical activity variables were evaluated using the Flemish Physical Activity Computerized Questionnaire (FPACQ). The FPACQ was found to be a reliable and valid questionnaire for the assessment of different dimensions of physical activity during a usual week in employed/unemployed and retired people. The FPACQ was used to calculate the physical activity level (PAL) of the NV subjects [18]. 

Nutritional intake data were collected using the same three-day food record for the V and the NV subjects. All diaries contained an instruction manual including portion size or weight or volume of common household measures. Volunteers were asked to weigh the consumed food items when possible or otherwise to indicate portion or size of the consumed food items. Analysis of the food records was performed with the BECEL program using the Belgian nutrient composition table [19]. 

### 2.3. Data Analysis

Statistical analyses were performed using SPSS. The significance level was set at 0.05. A chi-square test was used to compare the nominal data between the V subjects and the NV subjects. The data at scale level were compared using an independent sample t-test for comparisons between the two groups and one sample t-test for comparisons with the Belgian RDA after testing for normality (Kolmogorov Smirnov Goodness of Fit test). Since different Belgian reference values [20] exist for men and women, these groups were analyzed separately.

## 3. Results

### 3.1. Matching

No significant differences were found when comparing age, BMI and energy requirement between the V and NV subjects (Table 1).

**Table 1:** Age, BMI and energy requirement of the vegetarian (V) and the omnivorous (NV) subjects.

	Dietary pattern	Mean ± SD	p
***Age (years)***			
men (n = 35)	V	39 ± 14	0.709
	NV	40 ± 14	
women (n = 71)	V	35 ± 12	0.552
	NV	36 ± 12	
***BMI (kg/m^2^) ***			
men (n = 35)	V	23 ± 4	0.233
	NV	24 ± 3	
women (n = 71)	V	22 ± 4	0.441
	NV	23 ± 3	
***Energy req. (kcal) ***			
men (n = 35)	V	3,266 ± 481	0.608
	NV	3,209 ± 450	
women (n = 71)	V	2,336 ± 228	0.754
	NV	2,349 ± 263	

No significant differences were found when comparing the smoking habits of both the male and female matched V and NV subjects. Alcohol use during the week was not significantly different between V and NV subjects in both genders. Similar results were found for alcohol use during the weekend (Table 2).

**Table 2:** Differences by gender in smoking habits and alcohol use during the week and at the weekend between the vegetarian (V) and the omnivorous (NV) subjects.

Gender		Smoking habits		Alcohol use: week		Alcohol use: weekend	
		Yes	No	Yes	No	Yes	No
***Men***	V (n = 35)	4	31	10	25	18	17
	NV (n = 35)	4	31	8	27	19	16
		χ^2^ < 0.001; p = 1.000		χ^2^ = 0.299; p = 0.584		χ^2^ = 0.057; p = 0.811	
***Women***	V (n = 71)	9	62	25	46	45	26
	NV (n = 71)	8	63	16	55	44	27
		χ^2^ = 3.008; p = 0.222		χ^2^ = 2.778; p = 0.096		χ^2^ = 0.030; p = 0.862	

After matching for the above-mentioned characteristics, the educational level of both groups was compared. Table 3 shows there was a higher mean educational level in the V compared to the NV subjects.

**Table 3:** Educational level (primary school degree, high school degree, college and/or university degree) in the vegetarian (V) and the omnivorous (NV) subjects.

Diet	Primary school degree	High school degree	College and/or university degree
V (n = 106)	1	30	75
NV (n = 106)	6	49	51
χ^2^ = 12.712; p = 0.002			

### 3.2. Dietary Intake

#### 3.2.1. Energy requirement and energy intake

The Belgian prevailing standard for energy intake is 2900 kcal/day for men and 2150 kcal/day for women. Results show that none of the four groups in this study (V men and women, NV men and women) reach these standards (Table 4). For all groups, a discrepancy between energy requirement (calculated from PAL values) and energy intake was noted. This resulted in a significantly negative energy balance for all groups.

**Table 4:** Total energy and macronutrient intake (energy percentage, E%) in vegetarian (V) and omnivorous (NV) subjects.

		Men		Women	
	Ref.	V	NV	V	NV
		(n = 35)	(n = 35)	(n = 71)	(n = 71)
Total energy intake (kcal)	♀2,150	2,346 ± 685 ^†^	2,628 ± 632 ^†^	1,991 ± 539 ^†^	1,973 ± 592 ^†^
	♂2,900				
Total energy requirement (kcal)		3,266 ± 481^ †,a^	3,209 ± 450 ^†,a^	2,336 ± 228 ^†,a^	2,349 ± 263 ^†,a^
Protein (E%)	10-15	12.67 ± 2.32	15.29 ± 4.51 ^*^	13.23 ± 2.25	16.02 ± 3.98 ^*,†^
Carbohydrates (E%)	55-75	55.27 ± 10.09	47.40 ± 6.91 ^*,†^	55.08 ± 7.62	47.21 ± 8.19 ^*,†^
Mono & Disaccharides (E%)	<12	23.92 ± 9.93 ^†^	12.51 ± 5.18 ^*^	24.94 ± 7.35 ^†^	11.84 ± 6.60 ^*^
Polysaccharides (E%)		29.82 ± 6.87	24.78 ± 5.65 ^*^	27.64 ± 7.10	23.63 ± 6.51 ^*^
Fiber (g)	>30	34.55 ± 12.56	20.88 ± 7.92 ^*,†^	31.92 ± 12.90	16.29 ± 6.46 ^*,†^
Fat (E%)	15-30	29.30 ± 8.37	33.77 ± 5.25 ^*,†^	29.69 ± 6.91	34.66 ± 8.99 ^*,†^
Saturated fat (E%)	<10	10.17 ± 4.29	12.58 ± 2.97 ^*,†^	9.44 ± 3.06	12.87 ± 3.73 ^*,†^
Poly unsaturated fat (E%)	5.3-10	6.99 ± 2.31	5.92 ± 1.79 *	6.99 ± 2.83	5.72 ± 2.28 ^*^
P/S ratio	0.5-1.0	0.88 ± 0.72	0.50 ± 0.20 ^*^	0.76 ± 0.33	0.47 ± 0.18 ^*^
Mono unsaturated fat (E%)	>10	9.45 ± 2.80	12.86 ± 3.03 ^*^	9.99 ± 3.63	13.33 ± 4.82 ^*^
Alcohol (E%)	<4	3,4 ± 5,4	3,5 ± 5,4	2,8 ± 4,2	2,1 ± 4,2

^* ^Significantly different between V and NV (p < 0.05) ^†^ Significantly different to reference value (Ref.) (p < 0.05) ^a^ Significantly different to total energy intake (p < 0.05) P/S ratio: Poly unsaturated fat/Saturated fat ratio

#### 3.2.2. Energy distribution

The results in Table 4 demonstrate that both the V and the NV subjects reach the reference values for protein intake. However, the V have a significantly lower intake of proteins when compared to the NV, for both the men and the women. In addition, NV women have a protein value significantly above the RDA.

Carbohydrate intake (total, mono-, di- and polysaccharides) and fiber intake of both the V men and women is significantly higher when compared to the NV group. The V subjects meet the reference values for carbohydrate and fiber intake, whilst the carbohydrate and fiber intake of the NV subjects is too low.

V subjects meet the reference values for fat intake, whilst the fat intake of the NV subjects is too high. The V have a significantly lower intake of total and saturated fat when compared to the NV, for both the men and the women. The poly unsaturated fat intake is lower in the NV compared to the V subjects. This results in a more favorable poly unsaturated fat/saturated fat ratio in V. The mono unsaturated fat intake is higher in the V compared to the NV, for both the men and the women. The matching of V and NV subjects for alcohol use based on the general questionnaire is confirmed by the results of the three-day food record: no significant differences were found between V and NV for alcohol intake (Table 4).

#### 3.2.3. Minerals and vitamins

The intake of most minerals was significantly different between the V and the NV subjects. Sodium intake was lower in V men, potassium intake was higher in V women, calcium intake higher in V women, magnesium intake was higher in V women, zinc intake higher in V men and women, and iron intake higher in V men and women. β-carotene intake was higher in the V while the vitamin A intake was higher in NV. The vitamin C intake was comparable (Table 5).

**Table 5:** Mineral and vitamin intake of the vegetarian and the omnivorous subjects by gender (mean ± SD).

	Men			Women		
		Vegetarians	Omnivores		Vegetarians	Omnivores
	Ref.	(n = 35)	(n= 35)	Ref.	(n = 71)	(n = 71)
Sodium (mg/day)	575-3,500	2,711 ± 1043	3,388 ± 1,039 ^*^	575-3,500	2,728 ± 1654	2,749 ± 1,253
Potassium (mg/day)	2,000-4,000	3,620 ± 1068	3,563 ± 1,114	2,000-4,000	3513 ± 1479	995 ± 983 ^*,†^
Calcium (mg/day)	900	968 ± 451	795 ± 297 ^†^	900	964 ± 374	775 ± 362 ^*,†^
Phosphorus (mg/day)	800	1,476 ± 495	1,587 ± 483	800	1,336 ± 520	1,218 ± 401
Magnesium (mg/day)	420	439 ± 179	321 ± 110 ^†^	330	428 ± 347	253 ± 90^ *,†^
Iron (mg/day)	9	16 ± 6	14 ± 4 ^*^	8 (20a)	14 ± 6	11.7 ± 4.5 ^*^
Zinc (mg/day)	9.5	9.2 ± 3.7	4.7 ± 2.9 ^*,†^	7	8.3 ± 3.0	3.6 ± 2.3 ^*,†^
Vitamin A (µg/day)	600	830 ± 474 ^†^	3,354 ± 7,418 ^*,†^	500	855 ± 406 ^†^	3,393 ± 7,894 ^*,†^
β-carotene (µg/day)		2,799 ± 2,588	533 ± 637^ *,†^		3,150 ± 2,208	536 ± 530 ^*,†^
Vitamin C (mg/day)	110	116 ± 108	111 ± 108	110	107 ± 94	106 ± 96

^a ^during menstruation * Significantly different to vegetarians † Significantly different to reference value (Ref.)

## 4. Discussion

Studies have pointed out that the effect of a vegetarian diet and a higher intake of plant based food sources may be social class specific [21,22]. In this study, both groups had a high educational level, although the V subjects had an even higher educational level compared to the NV subjects. As it is the case in most of the study designs with self selected subjects, a possible selection bias regarding to the most health conscious ones (V and NV) and the most convinced vegetarians may have occurred. A possible explanation for the differences in educational level between these matched samples can be found in a study of Gale *et al.* (2007), who concluded that vegetarianism may be viewed by those of higher intelligence as a healthier option than consuming meat and that higher scorers for IQ in childhood are associated with an increased likelihood of vegetarianism in adulthood [23]. Results demonstrate that the energy requirement surpasses the energy intake. No significant differences were found between the energy intake of the V and the NV, for both genders. As reported in other studies [24,25], underreporting of energy intake and over reporting of physical activity may be a possible explanation for the discrepancies between energy intake and energy requirement in both V and NV subjects. The discrepancies between energy intake and energy requirement were of the same magnitude for the V and the NV despite a different method for the estimation of the physical activity level. However the Baecke questionnaire and the FPACQ are validated methods for the estimation of the PAL [17,18]. As no “gold standard” exists to estimate energy intake and requirement, a food record diary and the physical activity level may be considered the best possible way of matching these two samples [24,25]. Both V and NV subjects met the reference values for protein intake (with the exception that the NV women had slightly too high protein intake). However, the V had a significantly lower intake of proteins when compared to the NV, for both the men and women. A vegetarian diet suggests a higher intake of plant-based protein sources. An issue that is still unclear is whether all sources (plant based or animal) have the same impact on disease outcomes. A study of Keleman *et al.* [26] indicated that plant proteins have a more protective effect against coronary heart disease mortality compared with animal proteins, whereas no clear association with cancer incidence and mortality was observed for any subtype of protein. Other studies suggest that the higher protein consumption from animal sources is responsible for an acidifying effect in omnivores, which may increase the risk for compensation from the calcium sources of the body [13,27,28].

Carbohydrate intake of both the V men and women is significantly higher when compared to the NV group. Carbohydrates should deliver 55-75 E% of the total energy intake with no more than 12 E% originating from mono- and disaccharides (except when originating from high fruit consumption) and the rest from polysaccharides [20]. Both male and female V subjects had a high mono- and disaccharide intake, while NV subjects had an intake around 12 E%. However, this higher intake can be explained by higher fruit consumption in plant-based diets. A more in depth analysis on a subsample of these groups (V and NV) showed a higher fruit and vegetables intake in the V (respectively 283 ± 60 g/day and 268 ± 45 g/day) compared to the NV (120 ± 65 g/day and 185 ± 38 g/d, respectively) [13]. The V subjects had a significantly lower intake of total and saturated fat when compared to the NV, for both the men and the women. In addition, the results of the V are closer to the Belgian RDA than the results of the NV. Studies suggest that a lower dietary intake of saturated fat in V contributes to a reduction of ischemic heart diseases [7,27]. The higher saturated fat intake and lower poly unsaturated fat intake in the NV results in a better poly unsaturated fat/saturated fat ratio in V [20]. Appleby *et al.* [30] found a significantly inverse association between dietary fiber and BMI, and a significant positive association between animal fat intake and BMI. Alewaeters *et al.* [14] suggested that the lower BMI values in the vegetarian sample compared with the Belgian references may be a result of a healthier diet. Even with groups matched for BMI, a higher fiber intake and a lower saturated fat intake was found in the V compared to the NV subjects.

Significant differences in mineral intake were found between the V and the NV subjects. Sodium intake was lower in V men, and potassium, calcium and magnesium intake was higher in V women. Zinc and iron intake was higher in both V men and women. These minerals are often described as critical in a vegetarian diet [1,31]. Most of the iron intake in a vegetarian diet is non-heme iron, which is more difficult to absorb compared to heme-iron. However, the higher iron intake in the V combined with a plant-based diet, commonly rich in vitamin C, may improve the iron absorption [32]. β-carotene intake was higher in the V, whereas vitamin A intake was higher in NV. The vitamin C intake was comparable and met the RDA. The health aspects of daily fruit and vegetables intake are sustained by several epidemiological studies. Fruit and vegetables may protect against excessive weight gain because of their low energy density, high fiber content, low glycemic index and for bone health because of their alkaline-forming properties [33,34]. As reported in the Dietary Approaches to Stop Hypertension study, the blood pressure lowering effect of a vegetarian diet may be due to a high potassium and magnesium content delivered by fruit and vegetables [35]. Our results in the female vegetarians corroborate this finding.

In most studies it is difficult to disentangle the effect of other health benefits due to the vegetarian diet as a result of other health related behaviors. However, in this study of matched samples-where V and NV were similar with regard to a wide variety of physical, lifestyle and social factors-the V appear to consume a diet closer to the Belgian RDA compared to the diet of the NV. These results corroborate the findings of the Belgian national food consumption survey where similar dietary habits were found in NV subjects. It was concluded that an improvement of the Belgian food pattern, which is mainly an omnivorous diet, is necessary for a better prevention of diet-related diseases [36]. We can conclude, within the limits of our sample, that the results of this study suggest that the vegetarian diet consumed in this group is adequate to sustain the nutritional demands in a better way than the omnivorous diet. This indicates a possible contribution of a vegetarian or mainly plant based diet to an improvement of the Belgian food pattern for a better prevention of diet related diseases [1,36,37]. The care to ensure that a vegetarian diet is well planned has been recognized in many professional instances [1].

## References

[1] Craig W.J., Mangels A.R. (2009). Position of the American Dietetic Association: Vegetarian Diets. J. Am. Diet. Assoc..

[2] Chang-Claude J., Frentzel-Beyme R. (1993). Dietary and lifestyle determinants of mortality among German vegetarians. Int. J. Epidem..

[3] Key T.J., Fraser G.E., Thorogood M., Applby P.N., Beral V., Reeves G., Burr M.L., Chang-Claude J., Frentzel-Beyme R., Kuzma J.W., Mann J., McPherson K. (1999). Mortality in vegetarians and nonvegetarians: detailed findings from a collaborative analysis of 5 prospective studies. Am. J. Clin. Nutr..

[4] Appleby P.N., Thorogood M., Mann J.I., Key T.J. (1999). The Oxford vegetarian study: an overview. Am. J. Clin. Nutr..

[5] Leblanc J.Ch., Yoon H., Kombadjian A., Verger Ph. (2000). Nutritional intakes of vegetarian populations in France. Eur. J. Clin. Nutr..

[6] Davey G.K., Spencer E.A., Appleby P.N., Allen N.E., Knox K.H., Key T.J. (2003). EPIC-Oxford: lifestyle characteristics and nutrient intakes in a cohort of 33883 meat-eaters and 31546 non meat-eaters in the UK. Publ. Hlth. Nutr..

[7] Key T.J., Appleby P.N., Russell M.S. (2006). Health effects of vegetarian and vegan diets. Proc. Nutr. Soc..

[8] Key T.J., Appleby P.N., Spencer E.A., Travis R.C., Allen N.E., Thorogood M., Mann J.I. (2009). Cancer incidence in British vegetarians. Br. J. Cancer..

[9] Craig W.J. (2009). Health effects of vegan diets. Am. J. Clin. Nutr..

[10] Whitten C., Sabate J. (2001). Developing a vegetarian food guide. Vegetarian Nutrition.

[11] Nathan I., Hackett A.F., Kirby S. (1996). The dietary intake of a group of vegetarian children aged 7-11 years compared with matched omnivores. Brit. J. Nutr..

[12] Clarys P., Deriemaeker P., Hebbelinck M. (2000). Study of physical fitness and health related parameters in vegetarian and non-vegetarian students. Nutr. Food Sc..

[13] Deriemaeker P., Aerenhouts D., Hebbelinck M., Clarys P. (2010). Nutrient Based Estimation of Acid-Base Balance in Vegetarians and Non-vegetarians. Plant Foods Hum. Nutr..

[14] Alewaeters K., Clarys P., Hebbelinck M., Deriemaeker P., Clarijs J.P. (2005). Cross-sectional analysis of BMI and some lifestyle variables in Flemish vegetarians compared with non-vegetarians. Ergonomics.

[15] Duvigneaud N., Wijndaele K., Matton L., Deriemaeker P., Philippaerts R., Lefevre J., Thomis M., Duquet W. (2006). Prevalence of overweight, obesity and abdominal obesity in Flemish adults. Arch. Publ. Hlth..

[16] Demarest S., Van der Heyden J., Gilse L., Buziarsist J., Miermans P.J., Sartor F., Van Oyen H., Tafforeau J. (2002). Health Survey by Means of Interview, Belgium 2001.

[17] Baecke J.A., Burema J., Frijters J.E. (1982). A short questionnaire for the measurement of habitual physical activity in epidemiological studies. Am. J. Clin. Nutr..

[18] Duvigneaud N., Wijndaele K., Matton L., Philippaerts R., Lefevre J., Thomis M., Delecluse C., Duquet W. (2007). Dietary factors associated with obesity indicators and level of sports participation in Flemish adults: a cross-sectional study. Nutr. J..

[19] NUBEL (2004). Belgische Voedingsmiddelentabel.

[20] Hoge Gezondheidsraad (Superior Health Council) (2006). Voedingsaanbevelingen voor België (Nutritional recommendations for Belgium) dossiernr.

[21] Hulshof K.F., Brussaard J.H., Kruizinga A.G., Telman J., Lowik M.R. (2003). Socio-economic status, dietary intake and 10 y trends: the Dutch National Food Consumption Survey. Eur. J. Clin. Nutr..

[22] Herbert J.R. (1985). Relationship of vegetarianism to child growth in south India. Am. J. Clin. Nutr..

[23] Gale C.R., Deary I.J., Schoon I., Batty G.D. (2007). IQ in childhood and vegetarianism in adulthood: 1970 British cohort study. BMJ.

[24] Willett W.C. (1998). Nutritional Epidemiology.

[25] Prince S.A., Adamo K.B., Hamel M.B., Hardt J., Connor G.S., Tremblay M. (2008). A comparison of direct versus self-report measures for assessing physical activity in adults: a systematic review. Int. J. Beh. Nutr. Phys. Act..

[26] Keleman L.E., Kushi L.H., Jacobs D.R., Cerhan J.R. (2005). Associations of dietary protein with desease and mortality in a prospective study of postmenopausal women. Am. J. Epidemiol..

[27] Vormann J., Goedecke T. (2002). Latent acidosis: overacidification as a cause of chronic diseases. The physiological acid-base equilibrium and preventive aspects of a rich diet in bases. Schweiz Zschr. Ganzheitsmedizin.

[28] Frassetto L., Morris R.C., Sellmeyer D., Todd K., Sebastian A. (2001). Metabolic acidosis as the usual systemic acid-base state in humans. Eur. J. Nutr..

[29] Mann J., Appleby P.N., Key T.J., Thorogood M. (1997). Dietary determinants of Ischaemic heart disease in health conscious individuals. Heart.

[30] Appleby P.N., Thorogood M., Mann J.I., Key T.J. (1998). Low body mass index in non-meat eaters: the possible roles of animal fat, dietary fibre and alcohol. Int. J. Obes..

[31] Dwyer J.T. (1991). Nutritional consequences of vegetarianism. Ann. Rev. Nutr..

[32] Hallberg L., Hulthén L. (2000). Prediction of dietary iron absorption: an algorithm forcalculating absorption and bioavailability of dietary iron. Am. J. Clin. Nutr..

[33] Tucker K.L., Hannan M.T., Kiel D.P. (2001). The acid-base hypothesis: diet and bone in the Framingham osteoporosis study. Eur. J. Nutr..

[34] Mullie P., Clarys P., De Ridder D., Deriemaeker P., Duvigneaud N., Hebbelinck M., Grivegnee A.R., Autier P. (2006). Breakfast frequency and fruit and vegetable consumption in Belgian adolescents A cross-sectional study. Nutr. Food Sci..

[35] Appel L.J., Moore T.J., Obarzanek E., Vollmer W.M., Svetkey L.P., Sacks F.M., Bray G.A., Vogt T.M., Cutler J.A., Windhauser M.M., Lin P.H., Karanja N.A. (1997). A clinical trial of the effects of dietary patterns on blood pressure. N. Eng.J. Med..

[36] Vandevijvere S., De Vriese S., Huybrechts I., Moreau M., Temme E., De Henauw S., De Backer G., Kornitzer M., Leveque A., Van Oyen H. (2008). The gap between food-based dietary guidelines and usual food consumption in Belgium, 2004. Public Health Nutr..

[37] Sabaté J., Wien M. (2010). Vegetarian diets and childhood obesity prevention. Am. J. Clin. Nutr..

